# Characterization of polycyclic aromatic hydrocarbons in the Great Lakes Basin using dreissenid mussels

**DOI:** 10.1007/s10661-021-09401-7

**Published:** 2021-11-20

**Authors:** Kimani Kimbrough, Annie Jacob, Seann Regan, Erik Davenport, Michael Edwards, A. K. Leight, Amy Freitag, Mary Rider, W. Edward Johnson

**Affiliations:** 1 National Ocean Service, National Oceanic and Atmospheric Administration, National Centers for Coastal Ocean Science, 1305 East West Highway, Silver Spring, N/SCI1MD 20910 USA; 2 Consolidated Safety Services, 10301 Democracy Lane, Suite 300, Fairfax, VA 22030 USA

**Keywords:** Laurentian Great Lakes, PAH, *Dreissenid* mussel

## Abstract

The National Oceanic and Atmospheric Administration (NOAA), National Centers for Coastal Ocean Science (NCCOS) Mussel Watch Program (MWP), conducts basin-wide monitoring and place-based assessments using *dreissenid* mussels as bioindicators of chemical contamination in the Laurentian Great Lakes. Polycyclic aromatic hydrocarbons (PAHs) body burden results for the period 2009–2018 were combined into one dataset from multiple MWP studies allowing for a robust characterization of PAH contamination. Patterns in PAH data were identified using descriptive statistics and machine learning techniques. Relationships between total PAH concentration in *dreissenid* mussel tissue, impervious surface percentages, and PAH relative concentration were identified and used to build a predictive model for the Great Lakes Basin. Significant positive correlation was identified by the Spearman’s rank correlation test between total PAH concentration and percent impervious surface. The findings support the paradigm that PAHs are primarily derived from land-based sources. Offshore and riverine locations had the lowest and highest median total PAH concentrations, respectively. PAH assemblages and ratios indicated that pyrogenic sources were more predominant than petrogenic sources and that PAHs at offshore sites exhibited relatively more weathering compared to inshore sites.

## Introduction

Polycyclic aromatic hydrocarbons (PAHs) are a ubiquitous suite of environmental contaminants, comprised of aromatic rings, produced mainly from incomplete combustion of organic materials during natural events or anthropogenic activities. PAHs are comprised of compounds with different molecular structures (alkylation, number of aromatic rings) and physical properties (molecular weight, vapor pressure, water solubility, octanol water partitioning coefficient), which in turn affect their fate and transport in the environment (Schwarzenbach et al., 1993). Overall, the molecular structure and associated physical properties make PAHs hydrophobic and persistent in aquatic environments (Neff, 1979; Schwarzenbach et al., 1993). Some PAHs are carcinogenic, mutagenic, or teratogenic, posing a risk to organism and human health (Neff, 1979). Monitoring of PAHs occurs due to their continued release into the environment, persistence, and toxicity (O’connor, 2002; Richman, 2011; Burniston et al., 2012).

PAHs, due to their hydrophobicity, tend to adhere to particulate matter and enter the aquatic environment by atmospheric deposition, runoff, or direct point source discharge (McElroy, 1989). Spatially, PAH concentrations typically decrease logarithmically with distance from sources and range by orders of magnitude in the environment (Kimbrough & Dickhut, 2006; Kimbrough et al., 2008; Neff, 1979). Near cities and urban clusters, automobile exhaust represents a primary source of PAHs to the environment, while in remote areas, atmospheric input is the major source of PAHs (Gordon, 1976; Pierce & Katz, 1975). Deposition of PAHs associated with vehicular exhaust primarily occurs along roadways linking elevated PAHs to impervious surfaces and urban areas (Harrison & Johnston, 1985; Hewitt & Rashed, 1990).

PAHs are a concern in the Great Lakes, which constitute a major portion of North America’s freshwater. Great Lakes sediment show an increase in PAH concentrations correlated with proximity to urban clusters, and areas with increased population density (Van Metre et al., 2000; Lima et al., 2005; Van Metre et al., 2010). In the Great Lakes region, coal tar sealant on roadways and parking lots is also a primary source of PAH in sediments and represents another source of PAHs that are associated with impervious surfaces and developed areas (Baldwin et al., 2016; Mahler et al., 2005; Van Metre & Mahler, 2010).

There is a relationship between PAH alkylation, molecular weight, and temperature of formation (Lima et al., 2005). The lower the temperature of formation the greater the percentage of alkylated and lower molecular weight PAH compounds. Higher temperatures of formation result in fewer substituted and a larger percentage of higher molecular weight PAH compounds. The parent/homologue relationship is useful for distinguishing petrogenic and pyrogenic sources, as is the low/high molecular weight compound ratio (Boehm, 2014). Distinct compound relationships also extend to sources of PAHs (coal tar sealant, oil, urban sediment, creosote, automobile emissions, and diagenic weathering). We explore these relationships by using relative concentration (compound concentration/total PAH concentration), to normalize each sample, for comparison (Shields et al., 2015).

Due to the episodic nature of runoff, point sources, and atmospheric deposition, bivalves that filter particles from water make a good matrix for contaminant monitoring of PAHs. Use of bivalves (caged and in situ) for contaminant monitoring is conducted worldwide, nationally and regionally, because they are sedentary and integrate the contaminant signal in the surrounding environment temporally and spatially (Goldberg et al., 1978; Kauss & Handy, 1985; Cantillo et al., 1998; Chase et al., 2000; Gewurtz et al., 2002; O’Connor, 2002; Monirith et al., 2003; Richman & Somers, 2010; Richman, 2011; Beyer et al., 2017; Kimbrough et al., 2018). Specifically bivalves filter water and particles from the water column, some of the contaminants associated with these matrices are ingested and retained, allowing temporal pulses of contaminants in the environment to be integrated by bivalves. Overall, the uptake process of hydrophobic contaminants in bivalves is thought to be a “passive diffusive process/equilibrium partitioning process” (Beyer et al., 2017). Bivalve monitoring also provides information with respect to the bioavailability of contaminants and indicates the potential trophic transfer to wildlife and ultimately humans. Bivalves have limited ability to metabolize PAHs, and are more tolerant to extended PAH exposures, continuing to filter water during deployment, making them ideal candidates for PAH monitoring in particular (Livingstone, 1992).

In the Great Lakes, NOAA’s Mussel Watch Program (MWP) has used *dreissenid* mussels since 1992 to monitor a wide suite of organic contaminants, including PAHs (Edwards et al., 2016; Kimbrough et al., 2014; Lauenstein et al., 1997). MWP conducted a basin-wide contaminant monitoring study and several place-based contaminant characterization studies using *dreissenid* mussels to address a Great Lakes Restoration Initiative (GLRI) Action Plan II Measure of Progress, “Identify emerging contaminants and assess impacts on Great Lakes fish and wildlife.” In addition, the MWP collaborated with the Environmental Protection Agency’s Great Lakes Fish Monitoring Program to obtain mussels from several offshore locations in all of the Great Lakes except Lake Superior. The data from these studies were combined to represent samples from heterogeneous waterbodies (harbor, bays, nearshore, and offshore). The combined dataset is comprised of approximately 50% monitoring sites and 50% place-based study sites. Combining all of the data into one dataset presented the opportunity to identify new information, derived from a larger spatial scale, using a descriptive statistical approach. Unsupervised statistical techniques, such as the random forest and cluster analysis, allowed us to see broad patterns in the data, identify concertation magnitude and relative concentration groups, and provide a basis for inferences. Supervised random forest used the relationship between *dreissenid* mussel PAH concentration magnitude and impervious surface to predict PAH concentration levels. Overall, machine learning approaches, such as random forest, were used to predict and characterize PAH data because of their ability to deal with departures from normality (Raschka & Mirjalili, 2019).

Mussel Watch data has previously served to provide evidence of contaminant presence, assessments of baseline contaminant conditions, and the ability to track contaminant concentration changes over time. In some cases, the MWP documented baseline contaminant concentration levels that later provided context for concentrations measured after natural and man-made environmental disasters such as Hurricanes Katrina and Rita, the attack on the World Trade Center, and oil spills (Johnson et al., 2008; Lauenstein & Kimbrough, 2007). This is also a dataset suitable for comparison of PAH concentrations pre-/post-remediation or restoration of aquatic environments (Stout & Graan, 2010). In this manuscript, we present information on PAHs relevant to the Great Lakes, using multiple statistical techniques to characterize data, identify patterns, predict levels of elevated PAH concentration, and provide context for comparison to past results.

## Material and methods

### Mussel sampling, deployment, and recovery

**Fig. 1:**
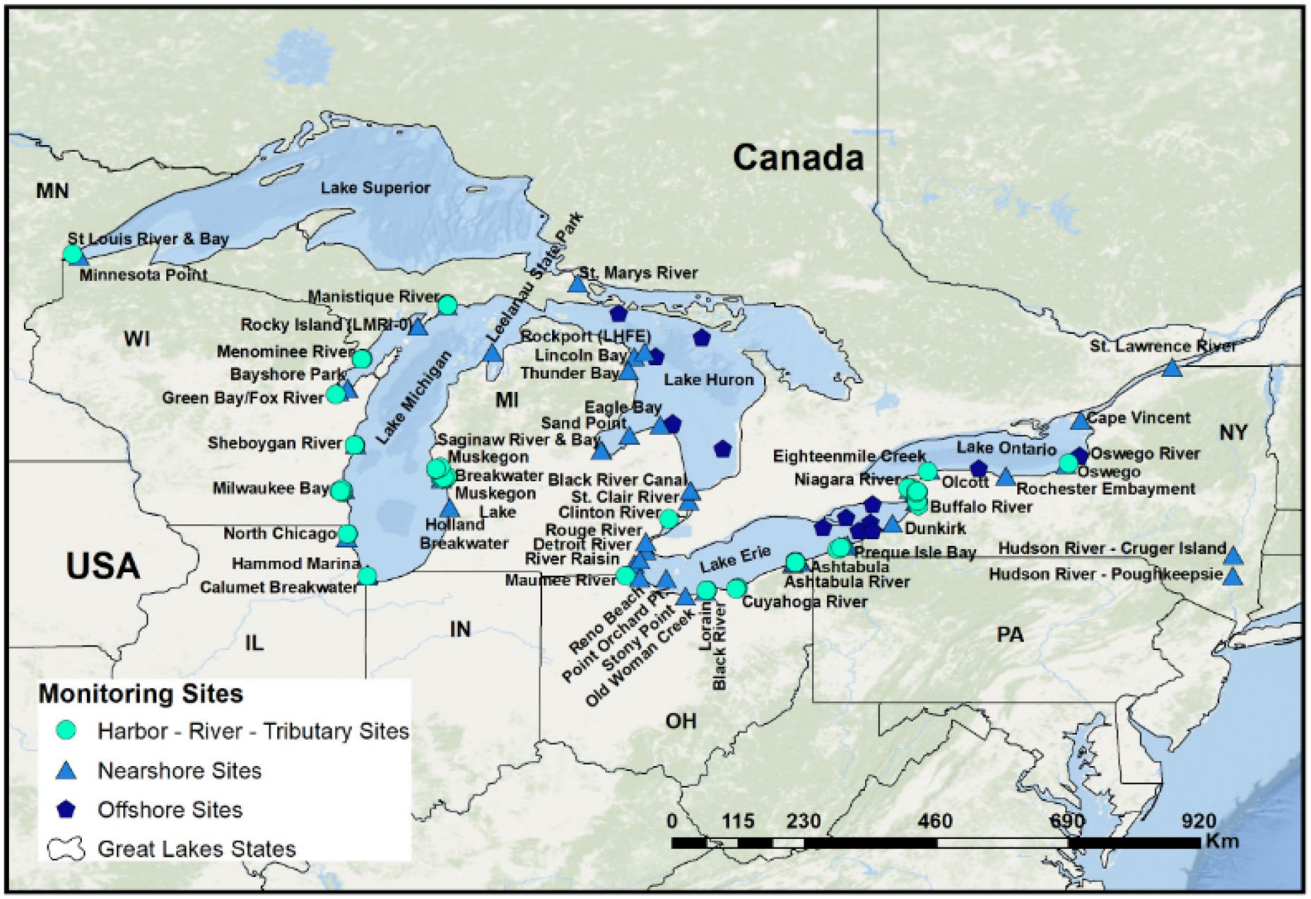
Great Lakes study locations occur throughout the basin. Some of the locations have multiple sites (supplemental information)

PAH concentrations were measured in *dreissenid* mussels collected in the Great Lakes from 2009 to 2018 (Fig. 1). The location of these sites includes offshore and nearshore lake sites; enclosed bays, including Green Bay, Presque Isle Bay, Muskegon Lake, and White Lake; and rivers/smaller tributaries. Two designations, inshore and offshore, were used to classify each site. Offshore sites included those samples taken in any of the Great Lakes or connecting channels that flow between/from them. Inshore samples include all samples taken from enclosed harbors, bays, and rivers. Samples were collected from urban and industrial locations such as Milwaukee, Cleveland, Ashtabula, Manistique, and Muskegon and from more rural areas such as Thunder Bay and Oswego (Kimbrough et al., 2014; supplemental information).

Mussel collection for the determination of PAH body burden followed two sampling approaches. The first approach, used primarily for lake and connecting channel samples, utilized in situ* dreissenid* mussels collected from hard substrates by diving, dredging, or ponar grab sampler. The second approach, used for multiple place-based contaminant assessment studies, utilized caged mussels deployed 4 or more weeks. Mussels respond to changes in environmental PAH concentrations (uptake and depuration) in less than 4 weeks, making our deployment time of 4 or more weeks acceptable. Studies have shown as little as 2 weeks may be needed to reach equilibrium (Beyer et al., 2017). Mussels from established populations on nearby stone breakwaters, in the nearshore, or harbor areas associated with each study were used to deploy cages. An initial time zero PAH concentration magnitude measurement was taken for mussels that were subsequently used for deployment in cages. Time zero mussel PAH concentrations did not show elevated concentration levels when compared to post-deployment caged mussel PAH concentration results. The time zero mussel PAH concentration was among the lowest concentrations and was always in the lowest cluster group relative to associated samples (supplemental information). Cages containing mussels, attached to moorings, resided one foot above the riverbed. After recovery, all mussels, including those from the harvest site, were packed on ice and shipped to a chemistry laboratory for analysis.

Laboratory preparation utilizes a minimum of 50–100 individual *dreissenid* mussels from one site that were shucked and homogenized. No size selection was used to preferentially obtain larger mussels. An aliquot of the homogenized mussel tissue sample was dried using Hydromatrix® followed by the addition of a surrogate standard (naphthalene-d8, acenaphthalene-d10, phenanthrene-d10, chrysene-d12, and perylene-d12) and accelerated solvent extraction. The extracts were cleaned using gel chromatography, followed by addition of internal standards (fluorine-d10, pyrene-d10, and benzo(a)pyrene-d12) before chemical analysis. Samples were then analyzed on a gas chromatagraph/mass spectrometer in select ion monitoring mode. More detailed protocols for organic contaminant analytical methods are found in Kimbrough et al. (2006) and Johnson et al. (2018). Measurements below the instrument method detection limit (MDL) were reported as zero for total PAH and PAH ratio calculations and were set to half the MDL for the unsupervised random forest analysis. All concentrations were blank corrected by subtracting blank concentration from samples concentration. Compounds associated with the deuterated naphthalene surrogate were not included due to low recoveries in some samples. To support comparisons with other studies, total PAH concentration magnitude was calculated by summing fifteen commonly reported parent compounds, while the unsupervised random forest analyses included all measured PAHs and heterocyclic compounds (Table 1).

**Table 1:** The Random forest analysis utilized all of the PAHs listed below to identify patterns in the chemistry concentration data

**Acenaphthylene (L)**	C1-Phenanthrenes_Anthracenes	**Chrysene (H)**
**Acenaphthene (L)**	C2-Phenanthrenes_Anthracenes	C1-Chrysenes
**Fluorene (L)**	C3-Phenanthrenes_Anthracenes	C2-Chrysenes
C1-Fluorenes	C4-Phenanthrenes_Anthracenes	C3-Chrysenes
C2-Fluorenes	Retene	C4-Chrysenes
C3-Fluorenes	Benzo[b]fluorene	**Benzo[b]fluoranthene (H)**
Benzothiophene	**Fluoranthene (H)**	**Benzo[k]fluoranthene (H)**
C1-Benzothiophene	**Pyrene (H)**	Benzo[a]fluoranthene (H)
C2-Benzothiophene	C1-Fluoranthenes_Pyrenes	Benzo[e]pyrene (H)
C3-Benzothiophene	C2-Fluoranthenes_Pyrenes	**Benzo[a]pyrene (H)**
C4-Benzothiophenes	C3-Fluoranthenes_Pyrenes	Perylene
Dibenzothiophene	C4-Fluoranthenes/Pyrenes	**Dibenzo[a,h]anthracene (H)**
C1-Dibenzothiophenes	Naphthobenzothiophene	C1-Dibenzo[a,h]anthracene
C2-Dibenzothiophenes	C1-Naphthobenzothiophene	C2-Dibenzo[a,h]anthracene
C3-Dibenzothiophenes	C2-Naphthobenzothiophene	C3-Dibenzo[a,h]anthracene
C4-Dibenzothiophenes	C3-Naphthobenzothiophene	**Indeno[1,2,3-c,d]pyrene (H)**
**Phenanthrene (L)**	C4-Naphthobenzothiophenes	**Benzo[g,h,i]perylene (H)**
**Anthracene (L)**	**Benz[a]anthracene (H)**	

The total PAH concentration magnitude was comprised of the bolded subset of parent PAHs. PAHs with parenthetic “L” are designated low molecular weight (LMW), and PAHs with parenthetic “H” are designated high molecular weight (HMW)

### Statistical analyses

Characterization of land use in proximity to each of the collection sites was achieved by developing 3000 m buffers around each site. The percentage of impervious surface within each buffer, based on the 2016 National Land Cover Database (NLCD), was then calculated. Although other buffer sizes were considered, the 3000 m buffer represented a tradeoff between local conditions and proximal land use for open water stations. Open water/offshore lake stations were the primary driver for using buffers. For PAHs, local sources and atmospheric deposition play major roles in determining site-specific PAH concentrations necessitating the use of buffers (Edwards et al., 2014, 2016). Buffers were used to characterize site proximity to impervious surfaces where automobiles and other PAH sources are found. Impervious surface data for each site was clustered to identify groups for characterization (Table 2). Sites near the Canadian border included small percentages of unquantifiable data due to the lack of compatible Canadian impervious surface data to combine with the NLCD coverage used in this study. These sites were primarily open water sites and Niagara River sites where the land use data within the buffer were less than 20% unidentifiable. Because the percent of unidentifiable land use was relatively small, or primarily water, these sites were included in the impervious surface analysis. Spearman’s rank correlation test characterized the relationship between impervious surface percentage and total PAH concentration.

**Table 2:** Dreissenid mussel total PAH tissue concentration magnitude (ng/g dry weight) descriptive statistics are presented for all data and by clusters (CL1-3), location (inshore/offshore), and impervious surface (clusters)

Categories	Groups	Mean	Median	Stdev	Min	Max	Count
All data		4913	919	9169	3	75,119	288
PAH concentration magnitude clusters	CL1	346	296	215	3	853	141
	CL2	2770	2414	1557	864	6145	92
	CL3	20,204	17,347	11,923	6855	75,119	55
Location	Inshore	7831	2789	10,968	27	75,119	171
	Offshore	647	388	879	3	5301	117
NLCD impervious surface cluster mean percentage	0–11	503	264	800	3	5301	78
	12–67	4420	1335	8547	28	75,119	176
	71–98	17,582	16,776	11,383	841	45,160	34

National Land Cover Database (NLCD) groups represent percent impervious surface ranges defined by cluster analysis and were used to compare PAH concentration magnitude results. ANOVA tests for PAH concentration magnitude cluster and impervious surface cluster were both significant (*p* < *.001*). All subsequent Tukey pairwise comparison tests for the ANOVAs were significant *p* < .002. *T* test results for the inshore/offshore comparison PAH concentration magnitude result were significant (*p* < .002)

PAH body burden data was combined into a single dataset from 288 *dreissenid* mussel samples collected between 2009 and 2018 by MWP (Fig. 1; supplemental information). The number of cluster groups for PAH magnitude was determined using the model-based clustering plots generated by the Mclust package in R (Fraley & Raftery, 2002; Fraley et al., 2012). Analysis of variance (ANOVA) was used to confirm differences in PAH magnitude associated with inshore/offshore sites, clusters, and impervious surface categories (Table 2).

Random forest, a multivariate ensemble learning method based on decision trees, was used to identify patterns in PAH data (Afanadora et al., 2016; Raschka & Mirjalili, 2019). Random forest is robust in handling outliers and correlated variables without decreasing prediction accuracy (Cutler et al., 2007), which is important for this data mining approach that used data collected from different MWP studies. All relative concentration PAH data was transformed using the package “scales” in R to mean center PAH relative concentrations before applying the unsupervised random forest method. Specifically, rows represented samples, and columns were designated for each PAH compound. The unsupervised analyses, performed with the R software, randomForest R package, used each compound’s relative concentration as a unique variable. The number of clusters was determined using the partition around medoids (PAM) from the cluster R package (Maechler, 2021). Together, random forest and PAM were used to identify patterns/clusters. Lastly, PAH magnitude concentration results, grouped by the unsupervised random forest/PAM cluster, were compared using ANOVA to confirm differences (Table 3).

**Table 3:** Dreissenid mussel total PAH tissue concentration magnitude (ng/g dry weight) descriptive statistics for random forest clusters used to group PAH concentration magnitude

Categories	Groups	Mean	Median	Stdev	Min	Max	Count
Random forest groups	RF1	1049	526	1563	129	13,731	93
	RF2	9648	4956	10,328	212	45,160	124
	RF3	1704	203	9166	3	75,119	71

ANOVA and Tukey pairwise analysis found all RF categories were significantly different (*p* < .002). This is evidence of the relationship between RF groups and concentration magnitude

After grouping and clustering PAH concentration magnitude results by inshore/offshore, concentration magnitude clusters, impervious surface clusters, and RF (relative concentration results), the impervious surface percentage data was used to predict PAH concentration magnitude clusters CL1-CL3 (Table 2). The createDataPartition package from the R caret package was utilized to create balanced train and test datasets (p = 0.80, set.seed = 150). The supervised random forest analysis (R randomForest) was used to predict PAH magnitude concentration clusters.

PAH ratios, derived from sample measurements, provided an opportunity to further characterize petrogenic (oil derived) and pyrogenic (combustion derived) PAHs. The specific ratios used in this paper, benzo[a]anthracene/chrysene, fluoranthene/pyrene, phenanthracene/anthracene, and pyrene/benzo[a]pyrene, were chosen because they were detected in most samples, and allowed for the greatest number of interpretable results. PAH ratios with concentrations of zero (below the detection limit) were excluded from our ratio analysis. A weight of evidence approach, including PAH ratios, pattern recognition, and visual assessment of charts, was used to identify patterns and provide an overall characterization of the PAH data.

## Results and discussion

### Total PAH spatial distribution

**Fig. 2:**
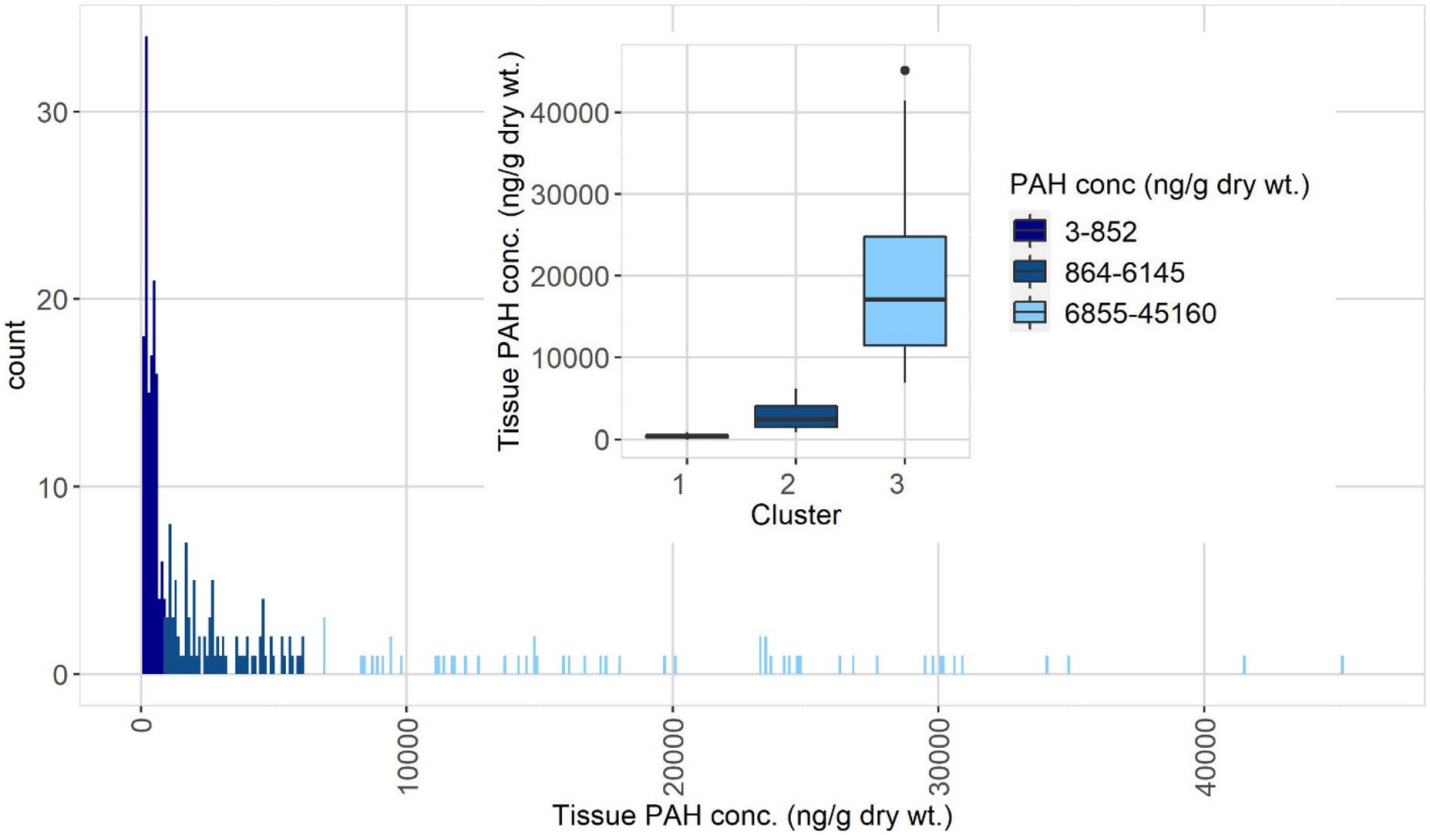
Histogram and box plot of the same PAH concentration magnitude data (same data as Table 2). For presentation clarity, one sample (75,119 ng/g dry weight) was excluded from both plots

Total PAH tissue concentrations from 288 sites in the Great Lakes Basin ranged several orders of magnitude (Table 2). A cluster analysis and subsequent ANOVA of total PAH magnitude concentrations identified three significantly (*P* < 0.002) different clusters (Table 2; Fig. 2). There was more than an order of magnitude difference in median total PAH concentrations between clusters CL1 and CL3 (Table 2; Fig. 2). This PAH data has a lognormal distribution similar to that found in data from the National Mussel Watch Program, composed primarily of low concentrations with the highest concentrations forming the tail of the distribution (Fig. 2; Kimbrough et al., 2008, 2014).

**Fig. 3:**
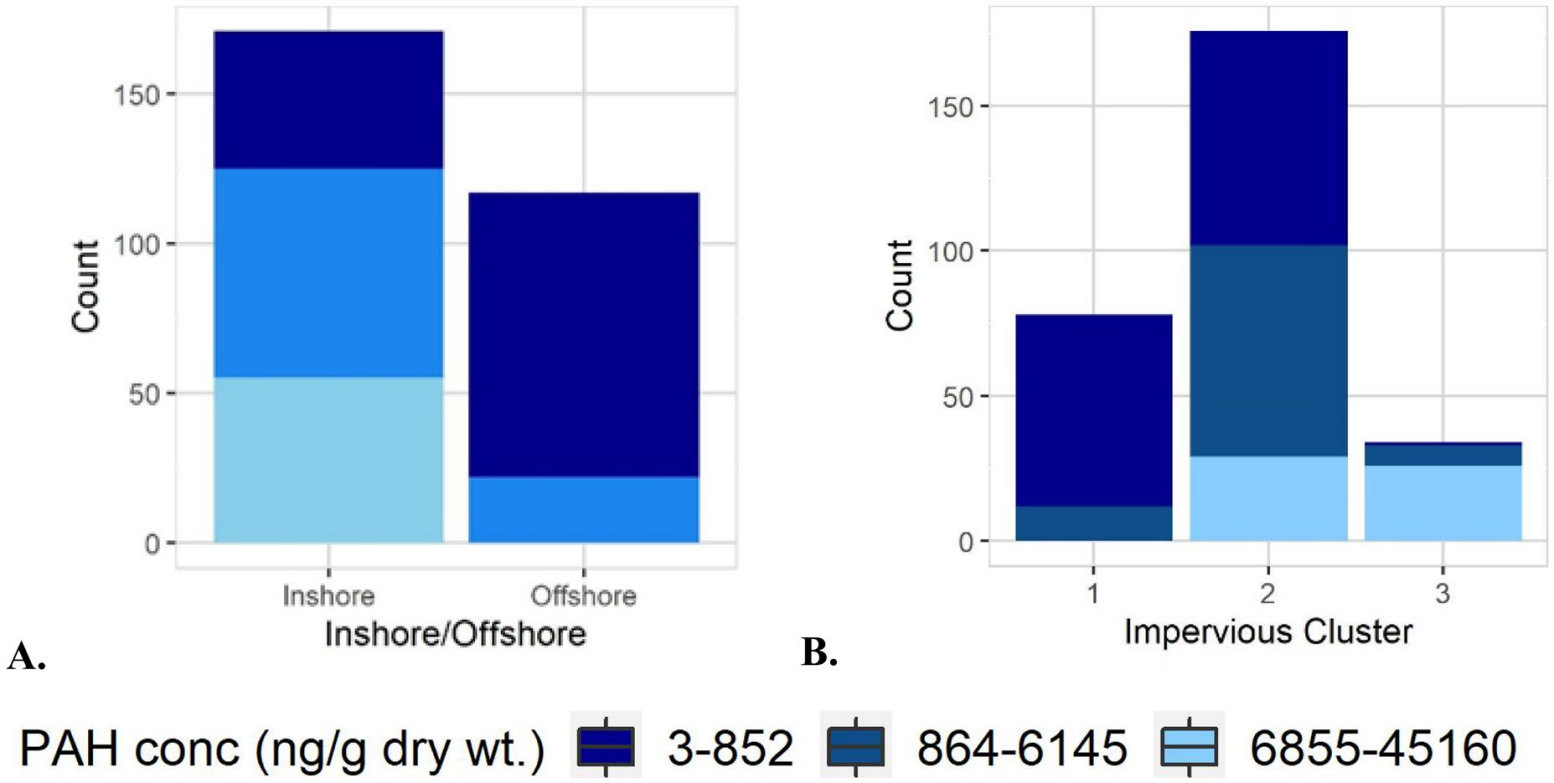
PAH magnitude group results from Table 2 are summarized by (**A**) inshore/offshore sites and (**B**) impervious surface clusters. For consistency, one sample (75,119 ng/g dry weight) was excluded from both plots

Analysis of inshore/offshore PAH concentrations using a *t* test found significant differences between inshore and offshore groups (*P* < 0.002). Median inshore total PAH concentrations were an order of magnitude higher than offshore median total PAH concentrations (Table 2; Fig. 3A). The offshore sites have a median total PAH concentration that was lower than the median for the entire dataset (Table 2), and lower than all but the first cluster (CL1), making offshore sites, as a group, among the lowest in this dataset, and thus substantiating their use as reference sites in bivalve health and comparison studies (Table 2).

Inshore sites had a PAH concentration magnitude with three orders of magnitude difference between minimum and maximum, including the highest concentrations. The cluster with the highest concentrations (CL3) was composed exclusively of inshore sites (Fig. 3A). Due to the small concentration range of offshore sites, from a monitoring perspective, fewer sites might be needed to characterize the lakes and connecting channels, which would be particularly important given the resource limitations of most monitoring programs. Care must be taken to include relevant baseline areas such as shipping channels and industrial sites in lakes for comparison in case of contaminant spills. However, this study supports the evidence that, with limited resources, monitoring efforts should focus on inshore areas (river, bays, and harbors), which are closer to sources and have far more variability. The higher variability in inshore data has a temporal component; sites sampled in different years or in different weeks may have different concentrations due to runoff events or releases from point sources. Proximity to sources (outfalls, waste water treatment plants) and dilution from lake water near the mouth of a river are additional sources of variability in samples from the same location. Another source of variability in samples from the same body of water is location; samples from the same body of water may be separated by meters or kilometers. As part of this study, proximity to sources such as outfalls or storm sewers was not quantified.

**Fig. 4:**
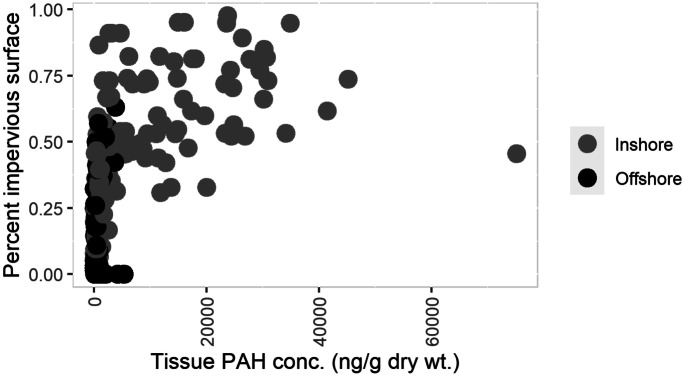
Spearman’s rank correlation identified a significant correlation between impervious surface percentage and total PAH concentration (p-value < 0.05, rho = 0.77)

Cluster analysis was used to group NLCD 2016 impervious surface results, resulting in three clusters (Table 2). ANOVA found significant differences (*P* < 0.002) in total PAH concentration between all impervious surface clusters (Table 2). Furthermore, a significant positive correlation was identified by the Spearman’s rank correlation test (rho = 0.77, p < 0.001) between total PAH concentration magnitude and percent impervious surface (Fig. 4). Urban settings, as shown in other studies, have point sources (power plants, industries) and non-point sources (automobile exhaust, road surface byproducts, and parking lot sealants) that can result in elevated PAH levels in aquatic systems (Krauss & Wilcke, 2003; Van Metre et al., 2000). Seal-coat and vehicle-related sources are primary sources of PAHs to urban lakes (Van Metre & Mahler, 2010) and represent a source of PAHs in developed areas. Offshore sites, which have less influence from land and increased influence from atmospherically derived PAH sources, are associated with lower total PAH concentrations. This diverse dataset derived from several studies identified total PAH concentration cluster/group differences associated with impervious surface percentage and location (Table 2). These groupings bring perspective to past PAH measurements and provide relevant information on the ranges of concentrations found in the Great Lakes Basin.

### Pattern recognition and PAH source characterization

**Fig. 5:**
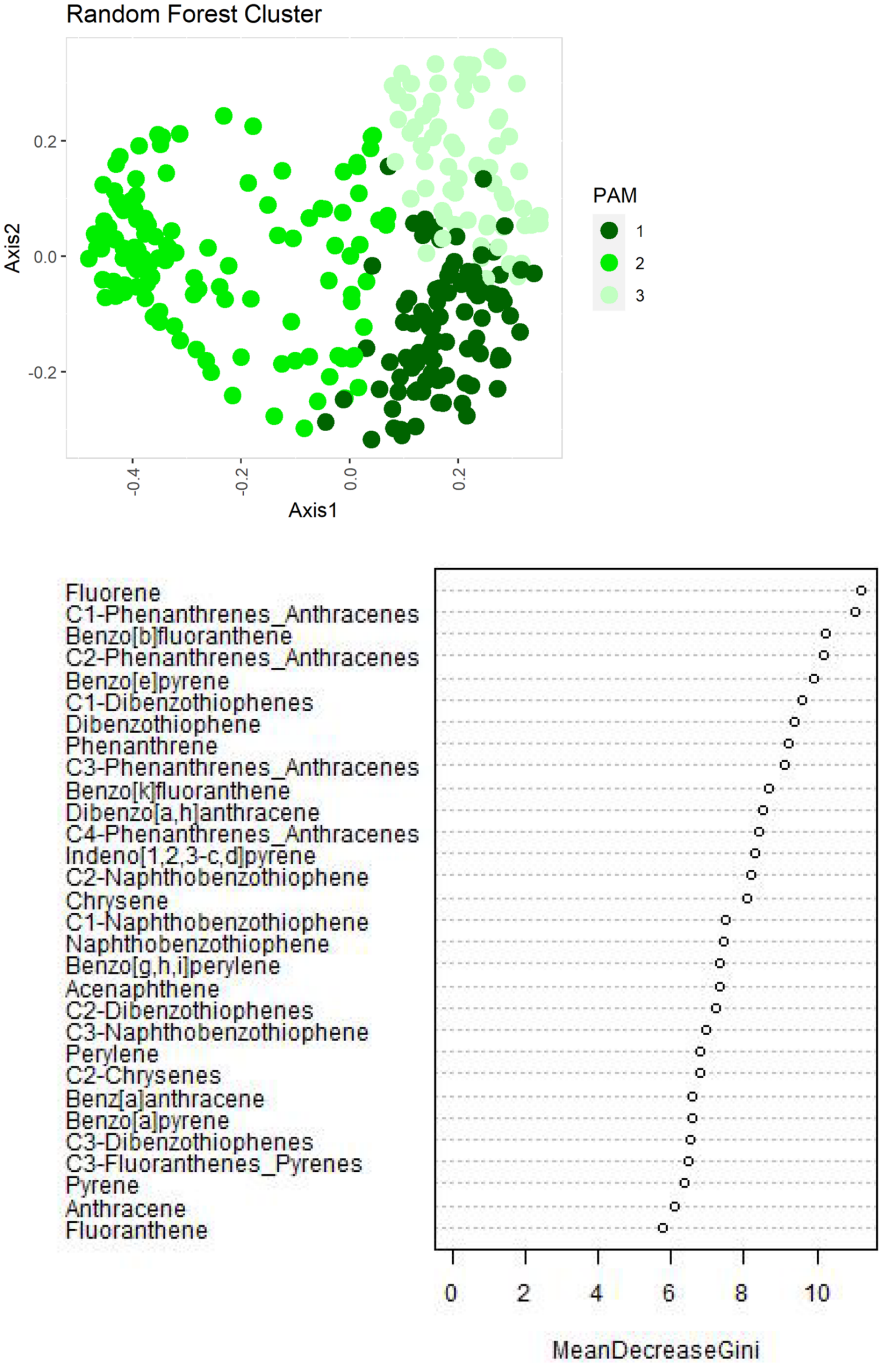
Axis 1 and 2 (top figure) represent the two variables from the unsupervised random forest analysis. Three clusters were identified in PAH relative concentration data using an unsupervised RF and partition around medoids (PAM) clustering (top). Each point on the plot represents a different sample/site. Mean Gini index was used to identify those relative compounds most responsible for distinguishing between the RF/PAM groups (bottom). The Gini index identifies/measures the importance of each compound to determining the various RF groups. The higher the score the more important the compound to forming the groups

**Fig. 6:**
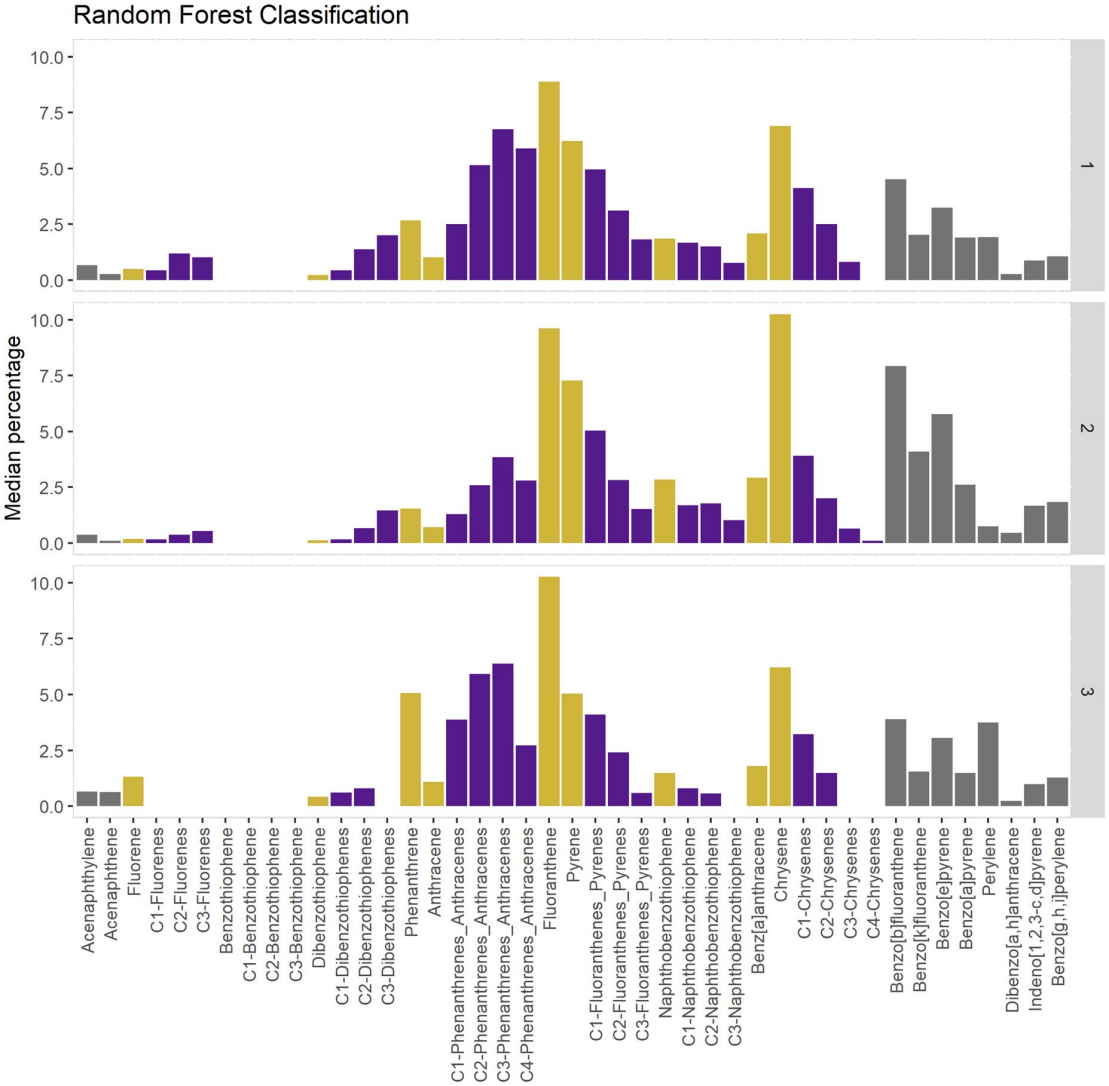
Relative concentration (compound concentration/total concentration) for PAH assemblages, using median values to characterize each random forest (RF) group (right axis). Petrogenic/pyrogenic origins of each RF group were characterized using parent and associated alkylated compounds (gold and purple, respectively). High and low molecular weight PAHs are defined in Table 1. All RF groups are predominantly pyrogenic; however, RF3 showed signs of weathering due to higher perylene concentration

**Fig. 7:**
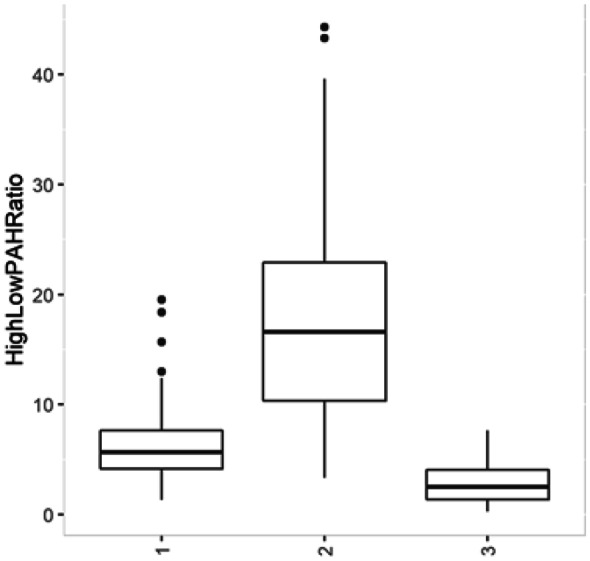
High to low PAH weight PAH ratios identified additional differences between the RF groups

Random forest (RF) analysis identified three clusters in PAH relative concentration data (Fig. 5). PAH relative concentrations were used to further characterize sources for each RF group. The relative proportion of high molecular weight (HMW) PAHs is greatest in RF2 (Fig. 6; Fig. 7). The presence of 4-ring and 5-ring HMW PAHs is indicative of a pyrogenic source in all RF groups (Lima et al., 2005). The high proportion of perylene observed in RF3 is indicative a diagenic sources. Specifically, perylene is a diagenic PAH generated by in situ changes that occur in the sediment (Hites et al., 1980; Venkatesan, 1988; Wakeham et al., 1980). Diagenic PAHs are not a primary component of anthropogenic PAH assemblages; however, when the overall anthropogenic signal is low (Table 3), their relative concentration may become locally relevant as seen in RF3 (Table 3; Fig. 6). Based on the more remote location of offshore sites, and absences of many 3-ring compounds, RF3 resembles a weathered and predominantly pyrogenic assemblage (Fig. 6).

**Fig. 8:**
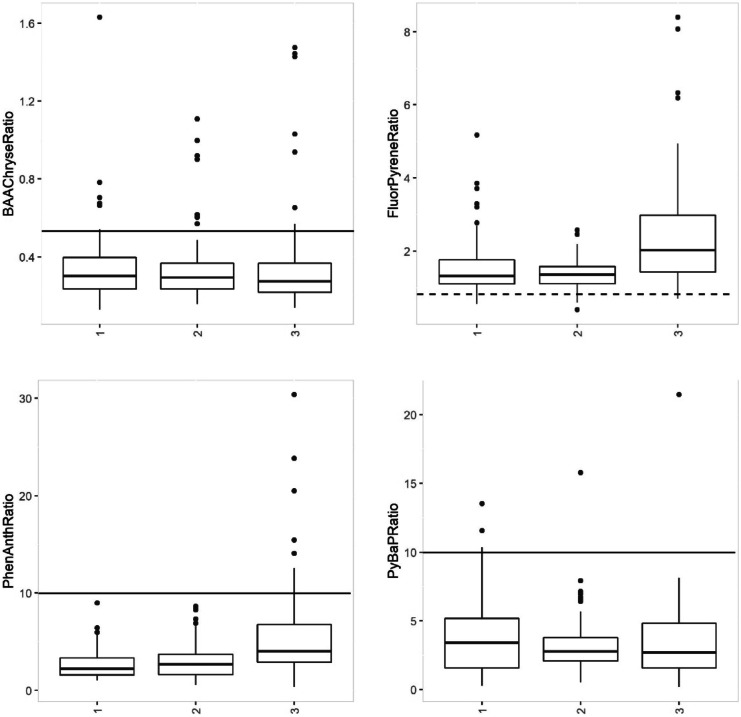
Petrogenic/pyrogenic origins of RF groups were characterized with PAH benz[a]anthracene /chrysene (BAAChryeRatio), fluoranthene/pyrene (FluorPyreneRatio), phenanthrene/anthracene (PhenAnthRatio), and pyrene/benzo[a]pyrene (PyBAPRatio) relative concentration ratios. The results support the overall conclusion of a predominantly pyrogenic source for all RF groups. Measurements below the solid lines and above the dotted line are indicative of various pyrogenic sources

Phenanthrene/anthracene (PA) ratios less than 10 are indicative of a pyrogenic source (Budzinski et al., 1997; Lima et al., 2005; Fig. 8). The majority of sites (97%) had PA ratios below 10. Nine offshore and one site located in Sturgeon Bay (3%) had PA ratios greater than 10, which could be the result of weathering or a petrogenic source (Fig. 8). Benz[a]anthracene/chrysene (BC) ratios below 0.5 are evidence of a pyrogenic automobile source (Dickhut et al., 2000; Gschwend & Hites, 1981; Fig. 8). Non-zero BC ratios below 0.5 comprised 91% of the results, indicating an automobile source contribution for the majority of sites. Fluoranthene/pyrene (FP) ratios greater than 1 are evidence of a pyrogenic source (Costa & Sauer, 2005; Gschwend & Hites, 1981). Eighty-eight percent of non-zero sites had FP ratios greater than 1, thus supporting the pyrogenic source interpretation of these results. Ninety-three percent of the sites had a pyrene/benzo[a]pyrene ratio (PB) less than 10, which is indicative of a pyrogenic source. The overall result for the four non-zero ratios all indicates that pyrogenic sources predominate (Fig. 8). Pyrogenic sources are associated with a higher percentage of high molecular weight PAH compounds relative to more petrogenic sources such as oil. However, in an environment with fewer chronic oil spills, we simply use this distinction to identify a difference rather than specific sources. Specific PAH source identification for major anthropogenic sources, such as coal tar sealants and automobile exhaust (Baldwin et al., 2016; Saha et al., 2009), was not attempted due too spatial and temporal extent of this study. Future source apportionment studies will be conducted at specific locations where more is known about local sources.

**Fig. 9:**
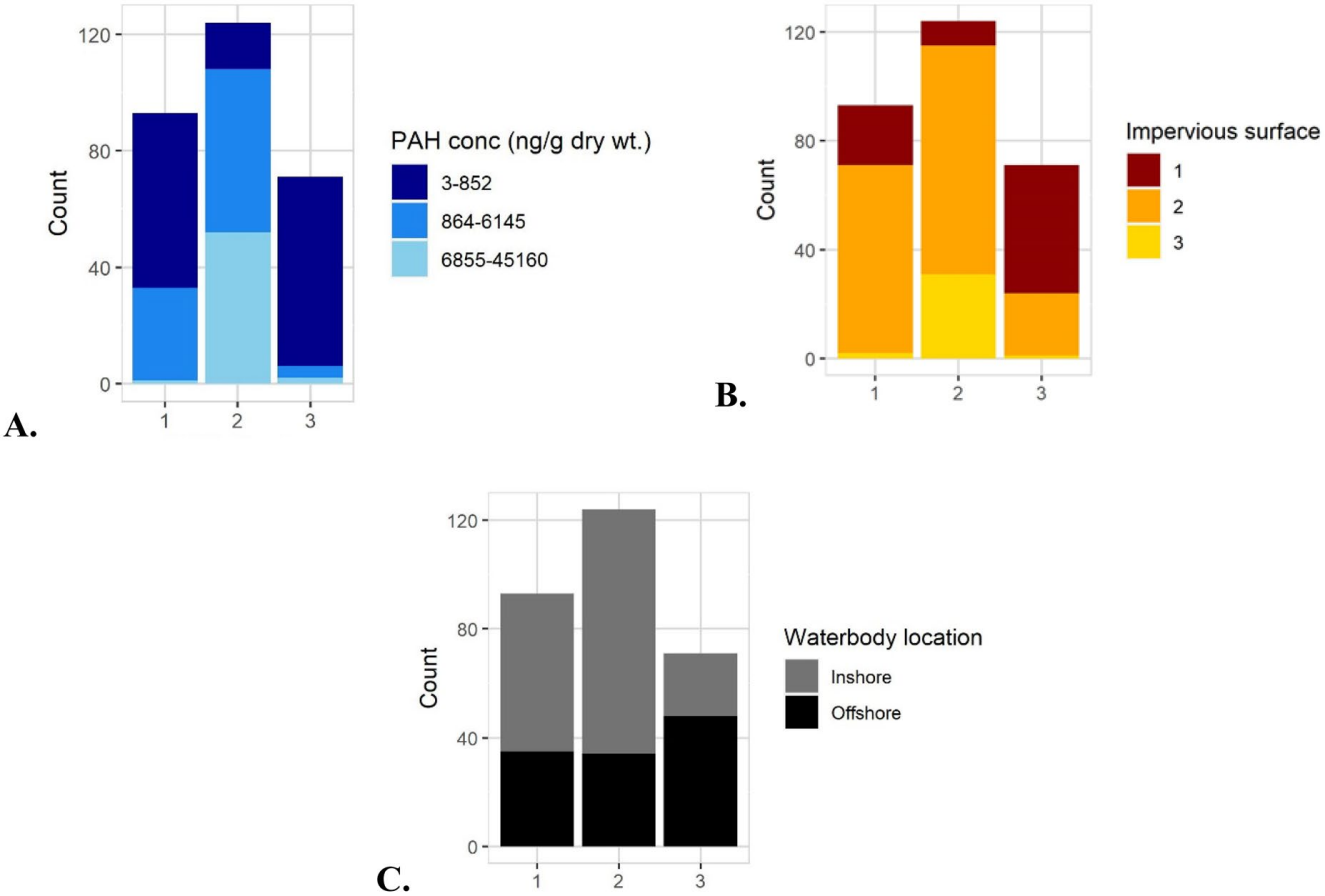
Independent variables were used to characterize each RF group (x-axis), with the most differences found between RF2 (elevated concentrations, and high impervious surface percentage) and RF3 (low concentration, and low impervious surface percentages). Mean impervious surface percentage for RF clusters 1, 2, and 3 were 29, 51, and 13, respectively. Overall, these results uncovered a relationship between relative concentration patterns, total PAH concentration, impervious surface percentage, and location. For consistency, one sample (75,119 ng/g dry weight) was exclude from plot A

Significant differences were also found between the RF groups with respect to PAH concentration magnitude and impervious surface. The magnitude of concentrations for all RF categories were significantly different (*P* < 0.002) from each other (Table 3). RF2 and RF1 represented the highest and lowest concentration magnitudes, respectively (Table 3; Fig. 9A). RF2 was comprised of more inshore sites and had a higher percentage of higher impervious surface sites relative to RF1 and RF3 (Fig. 9B, C). RF groups had differences in impervious surfaces, inshore/offshore, and overall total PAH concentration magnitude, thus highlighting the relationship between PAH composition (RF), waterbody location, PAH concentration magnitude, and impervious surface percentage (Fig. 9).

**Fig. 10:**
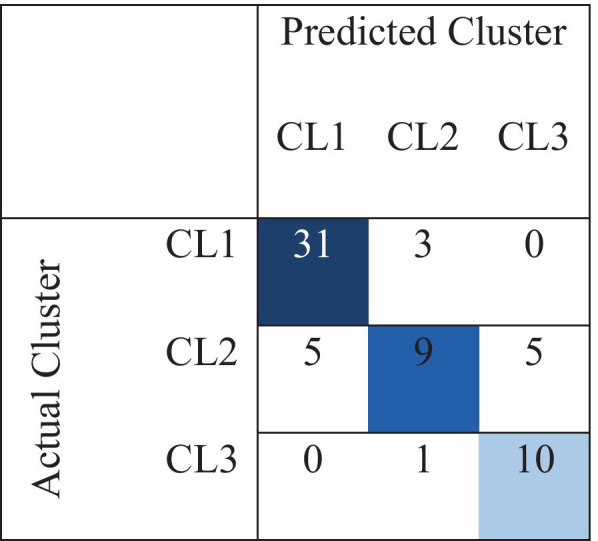
Confusion matrix for random forest prediction of concentration where 20% of samples were used for test/prediction. CL1, CL2, and CL3 represent 3–852, 864–6145, and 6855–75,119 ng/g dry weight respectively (Table 2). The prediction for the highest concentration cluster CF3 was ~ 90%, and the lowest concentration cluster CF1 was ~ 90% which were better than the middle concentration cluster CF2 (47%)

Finally, we were able to use the relationship between impervious surface and PAH magnitude to successfully predict two concentration cluster (Fig. 10). We used this method as we had more success predicting sites with elevated PAH concentration levels than to predicting actual PAH concentration. Due to the strong correlation between PAH concentration and impervious surface only one independent variable, impervious surface percentage was used for prediction. Specifically, CF1 and CF3 were predicted with approximately 90% accuracy (Fig. 10).

## Conclusions

Multiple years of data from different studies, with different sampling designs, were brought together to find new information at a larger spatial scale. Differences in concentrations between impervious surface clusters and site location (inshore, offshore) were identified. Specifically, lower concentrations were found offshore and in areas with lower percent impervious surfaces. Elevated PAH concentrations were found in urban rivers with high percent impervious surface, and positive correlation was identified between impervious surface percentage and total PAH concentration magnitude. The impervious surface and inshore/offshore results were not surprising, but the quantification of PAH concentrations in this large study provided *dreissenid* mussel measurements to bring perspective to future assessments.

Unsupervised random forest, used to find patterns in relative concentration data, identified three separate clusters of sites. The random forest clusters had significantly different concentrations, evidence of a relationships between PAH total concentration and PAH relative concentration. Furthermore, impervious surface was used to predict elevated and low *dreissenid* mussel PAH concentrations. Overall, this study was able to identify new information at a basin-wide scale; support the paradigm that PAHs are primarily derived from land-based sources; and identify pyrogenic PAH as the predominant sources of PAHs at the sites sampled. The high-level characterization of this data provides new information not available from a single site assessment.

## Data Availability

Upon publication this data will be made available on the National Centers for Coastal Ocean Sciences Great Lakes Mussel Watch project page (https://www.regions.noaa.gov/great-lakes/index.php/great_lakes-restoration-initiative/toxics/mussel-watch-expansion/), on the NOAA DIVER web portal (https://www.diver.orr.noaa.gov/), and as part of the supplemental information submission.
